# AR-12 Inhibits Chaperone Proteins Preventing Virus Replication and the Accumulation of Toxic Misfolded Proteins

**DOI:** 10.4172/2155-9899.1000454

**Published:** 2016-09-16

**Authors:** Laurence Booth, Jane L Roberts, Heath Ecroyd, St. Patrick Reid, Stefan Proniuk, Alexander Zukiwski, Abraham Jacob, Elsa Damonte, María J Tuñón, Paul Dent

**Affiliations:** 1 Department of Biochemistry and Molecular Biology, Virginia Commonwealth University, Richmond, VA 23298, USA; 2 School of Biological Sciences and Illawarra Health and Medical Research Institute, University of Wollongong, NSW 2522, Australia; 3 Molecular and Translational Science, United States Army Medical Research Institute of Infectious Diseases (USAMRIID), 1425 Porter Street, Fort Detrick, Frederick, MD 21702-5011, USA; 4 Arno Therapeutics, Flemington, NJ 08822, USA; 5 Department of Otolaryngology, The University of Arizona Ear Institute, 1515 North Campbell Avenue, PO Box 245024, Tucson AZ 85724, USA; 6 FCEN-UBA, Ciudad Universitaria, Pabellón 2 Piso 4, lab QB-17, 1428 Buenos Aires, Argentina; 7 Institute of Biomedicine and CIBEREhd, University of León, 24071, Spain

**Keywords:** Autophagosome, Phosphodiesterase, Tumor cell biology

## Commentary

Although originally thought to be an inhibitor of PDK-1, OSU-03012 (AR-12) was shown in 2005 *not* to primarily act as a PDK-1 inhibitor in its ability to radio-sensitize tumor cells [1,2]. Subsequently it was shown that the primary mechanism by which AR-12 killed tumor cells was via the PKR-like endoplasmic reticulum kinase (PERK) -dependent induction of endoplasmic reticulum stress signaling [1–3]. ER stress signaling in turn promoted a toxic form of autophagy resulting in a necroptotic form of cell death.

As AR-12 was causing an ER stress signal, the on-going studies became more focused on defining whether the functions and activities of chaperone proteins, particularly HSP90, HSP70 and GRP78, were being altered by the drug [3]. By western immuno-blotting, AR-12 lowered the expression of HSP90 and GRP78 but stimulated HSP70 expression. These findings were independently confirmed [4]. As AR-12 reduced expression of the PERK inhibitory chaperone GRP78, and as the induction of toxic autophagy was PERK dependent, we investigated in more detail the role of altered GRP78 expression in mediating drug toxicity. AR-12 destabilized GRP78 protein, significantly lowering its half-life as assessed by western blotting from >24 hours to 10 hours [5]. Transfection of cells with a plasmid to force over-expression of GRP78 blunted AR-12 induced PERK activation; autophagosome formation, and tumor cell death.

Data published in 2014 and 2015 with AR-12 have additionally underlined the prominence of chaperones and especially GRP78 in the cell biology of OSU-03012. Phosphodiesterase 5 inhibitors including sildenafil (Viagra) and tadalafil (Cialis) were shown to interact with OSU-03012 to facilitate killing in a wide variety of tumor cells. The enhanced killing effect of the drug combination was associated with enhanced PERK-dependent ER stress signaling and autophagosome formation, as well as through death receptor activation [6]. Congruent data were obtained using the parent drug of OSU-03012, celecoxib, and also with the multi-kinase inhibitors sorafenib, regorafenib and pazopanib [7,8]. These pre-clinical studies have resulted in two open clinical trials; in all solid tumor patients (NCT02466802) where patients are receiving increasing once daily dosing with regorafenib and sildenafil; in recurrent glioblastoma patients (NCT01817751) where they receive sorafenib, sildenafil and valproate twice daily.

From the research of many laboratories it has been defined that multiple chaperone proteins interact to play key roles in preserving protein stability and signaling, most notably in tumor cells which very often express much higher levels of protein than non-transformed cells. Hence some chaperones, e.g. HSP90, have become a target for developmental therapeutic synthetic chemists and also tumor cell biology scientists. In the area of viral reproductive biology, proteins such as HSPA5/ GRP78/ BiP have for almost 30 years been recognized as playing key facilitator roles in the life cycles of both DNA and RNA viruses [9–21].

Several years ago, we noted that our examination of chaperone functionality in the field of cancer developmental therapeutics would overlap with the findings from virology laboratories regarding the roles of chaperones in virus biology. From this realization we have performed studies to determine whether OSU-03012 could alter virus reproduction. OSU-03012 and the multi-kinase inhibitors sorafenib (Nexavar) and pazopanib (Votrient) reduced the expression of multiple chaperones in the HSP70 family and the HSP90 family; effects that were magnified by the PDE5 inhibitor sildenafil [7,22,23]. And, because our drug combinations were inhibiting the chaperone function of many diverse chaperones, we found that OSU-03012 or sorafenib or pazopanib could strongly inhibit the reproduction of antiviral a wide range of DNA and RNA viruses. We demonstrated that the reduction of GRP78 protein levels was one essential effect of these drugs in blocking virus reproduction. Contemporaneously with our studies, two groups of researchers demonstrated that the expression of GRP78 was essential for Ebola virus reproduction *in vitro* with molecular knock down of GRP78 *in vivo* protecting mice from Ebola virus lethality, and that OSU-03012 prevented the replication of hemorrhagic fever viruses *in vitro*, including Ebola and Marburg [24,25].

To further broaden the scope of our analyses, we implemented proteomic studies using OSU-03012 as bait [26]. The proteomic analyses revealed that chaperone and chaperone-regulatory proteins associated with OSU-03012: GRP75, HSP75, BAG2, HSP27, ULK-1, and thioredoxin.

The ATP binding site in HSP70 and HSP90 family chaperones is located in the NH2-terminal portion of the proteins and OSU-03012 rapidly reduced the *in situ* immuno-fluorescence detection of these chaperones using antibody epitopes against the NH2-termini of the proteins. Computer analyses demonstrated that OSU-03012 docked *in silico* with the ATPase domains of HSP90 and of HSP70. At the early time points very little alteration in the immuno-fluorescence signals were found using antibodies directed against epitopes in the central and COOH portions of the proteins. OSU-03012 altered the subcellular localization of chaperone proteins, eliminating their punctate stippled appearance at the same time as changing protein co-localization. OSU-03012 reduced chaperone ATPase activity, which was further facilitated by sildenafil. In agreement with all of these changes in biology OSU-03012 reduced chaperone – chaperone and chaperone – client interactions. OSU-03012 alone or when combined with sildenafil in a chaperone inhibitory fashion to strongly induce an eIF2α/ ATF4/ CHOP/ Beclin1 pathway in parallel with lowering mTORC1 and mTOR2 activities, and thus promoting increased ATG13 phosphorylation. All of these events together facilitated the formation of toxic autophagosomes. Over-expression of multiple dyad combinations of chaperone proteins prevented OSU-03012 –inducing ER stress signaling and also maintained mTOR activity with no change in P-ATG13 S318 levels.

In our 2016 virology-based publication we found that HSP90, HSP70, GRP78 and the small chaperone HSP27 are essential OSU-03012 effectors in terms of the drug changing viral biology [26]. Combined knock down of GRP78 and HSP27 profoundly lowered virus reproduction. The knock down of multiple chaperones or AR-12 exposure caused the expression of virus receptors and essential glucosidase proteins to be lowered. Combined knock down of chaperones or AR-12 treatment inactivated mTOR and at the same time elevated ATG13 S318 phosphorylation alongside inducing an ER stress response that enhanced Beclin1 and LC3 expression and ultimately autophagy. Cells over-expressing chaperone proteins blocked the reduction in receptor/glucosidase expression, mTOR inactivation, ER stress response and autophagy induction [27]. The reproduction of viruses as diverse as, and including: Mumps, Influenza, Measles, Junín, Rubella, HIV (wild type and protease inhibitor resistant), and Ebola was suppressed by AR-12. And, as noted previously, the impact of AR-12 on virus reproduction was replicated by chaperone protein knock down. AR-12 enhanced the co-localization of LC3 in autophagosomes with Influenza, EBV and HIV virus proteins. This effect was concomitant with reduced viral protein co-localization with the chaperones HSP90, HSP70 and GRP78. Beclin1 knock down lowered drug-enhanced autophagosome formation and lowered the anti-viral protection caused by AR-12. In an animal model of hemorrhagic fever virus, low doses of AR-12, for 24 hours, doubled animal survival from ~30% to ~60% and reduced liver damage as measured by ATL, GGT and LDH release [28,29]. Hence by inhibition of chaperone proteins; lowering the production, stability and processing of viral proteins; and promoting autophagosome formation/ viral protein break-down, AR-12 acts as a broad-specificity anti-viral drug *in vitro* and *in vivo*.

The actions of chaperones, including GRP78, have however also been linked to other human maladies, beyond viruses and cancer, to include neuro-degenerative disorders such as Alzheimer’s Disease [30–33]. GRP78 and other chaperones are over-expressed in the neurons of Alzheimer’s patients and they act to maintain cell viability and the correct tertiary conformation of tau protein and prevent formation of insoluble aggregates. Thus chaperone functionality in Alzheimer’s disease and the possibility of AR-12 or analogues of AR-12 being developed as a therapeutic for this disorder should be considered if supported by appropriate in vivo preclinical models. Of interest, AR-12 has been demonstrated to suppress neurite degeneration in an *in vitro* model of amyloid-β peptide (Aβ) 25–35-mediated neurite degeneration [32]. In patients who are initially presenting with mild initial memory loss and who presently have a low protein aggregate load in their neurons, the chaperone-inhibitory effects of AR-12 may result in a beneficial reduction in the amount of intracellular tau protein. AR-12 will cause an ER stress signal reducing the production of total cellular protein and simultaneously by de-chaperoning the tau proteins and promoting autophagy will further reduce the levels of tau protein. As brain-permeant PDE5 inhibitors are considered to be potentially useful agents in Alzheimer’s treatment and also enhance the chaperone inhibitory effects of AR-12; and as AR-12 crosses the blood-brain barrier in pre-clinical models; the development of AR-12 or AR-12 + PDE5 inhibitor therapy regimen for this debilitating disease should be considered. Also of interest, AR-12 has been demonstrated in an *in vitro* setting to have a very rapid and robust effect on decreasing PrP^Sc^ levels in prion-infected neuroblastoma cells (ScN2a). The rapid anti-prion effect was felt to result from increased PrP^Sc^ clearance consistent with the experimental data which demonstrated an up-regulation of autophagy markers including LC3-II and using ScN2a cells with CRISPR/Cas9-based autophagy knock-out (ATG5 gene) proved that autophagy is involved in the mode of anti-prion action of AR-12 [34].

In conclusion, we have shown that the drug OSU-03012 (AR-12), originally developed as an anti-cancer therapeutic, can be re-purposed as an anti-viral. AR-12 is not the only non-cancer drug we have recently repurposed and translated to the clinic. Dimethyl fumarate, ruxolitinib, celecoxib, valproic acid and sildenafil are medications for multiple sclerosis, myeloproliferative disorders, arthritis, bipolar disease and erectile dysfunction, respectively, and all in a rational manner can be combined with each other or with established cancer therapies to kill tumor cells. It is hoped that our initial studies in the area of repurposing, combined with those of other groups, may yield better and novel ways to treat cancer and viral diseases.

## Abbreviations

CAR: Coxsakie and Adenovirus Receptor
CD: Cluster of Differentiation
OSU: OSU-03012 also called AR-12
SIL: Sildenafil also called Viagra
TAD: Tadalafil also called Cialis
PTEN: Phosphatase and Tensin homolog
R: Receptor
dn: dominant negative
CMV: Empty vector control plasmid
COX: Cyclooxygenase
P: Phospho-
ca: constitutively active
WT: Wild Type
PERK: PKR like Endoplasmic Reticulum Kinase
HSP: Heat Shock Protein
GRP: Glucose Regulated Protein
